# Changes in TRPV1-Mediated Physiological Function in Rats Systemically Treated With Capsaicin on the Neonate

**DOI:** 10.3390/ijms21093143

**Published:** 2020-04-29

**Authors:** Keun-Yeong Jeong

**Affiliations:** MetiMedi Pharmaceuticals Co., Research Center, Incheon 22006, Korea; alvirus@naver.com; Tel.: +82-23080345

**Keywords:** neonatal capsaicin treatment, rat, TRPV1, pain, hyperthermia, infection, neuropathic itch, circadian rhythm

## Abstract

Capsaicin is the active component of chili peppers and is a hydrophobic, colorless, odorless, and crystalline to waxy compound. The transient receptor potential vanilloid 1 (TRPV1) is the capsaicin receptor channels that are involved in a variety of functions like transduction and transmission of the physiological stimulus. Subcutaneous injection of capsaicin to a newborn rat leads to involuntary lifelong TRPV1 desensitization. Various physiological changes including sensory and homeostatic actions in the body associated with neonatal capsaicin treatment are induced by direct TRPV1 channel targeting. Interesting changes include unique phenomena such as the reduction in pain perception, abnormal body temperature, increase in infection, infectious or neuropathological itching, and irregular circadian core body temperature rhythm. These symptoms are associated with relatively higher fever or loss of sensory c-fiber related to TRPV1 desensitization. The aforementioned outcomes not only provide a warning about the risk of capsaicin exposure in newborns but also indicate the possible occurrence of relatively rare diseases that are difficult to diagnose. Therefore, Therefore, the present review aims to summarize the unique phenomena caused by systemic capsaicin administration in neonatal rats.

## 1. Introduction

Capsaicin is an active ingredient of chili peppers which are plants belonging to the genus Capsicum. It is an irritant and stimulant for mammals, including humans, and generates a sensation of burning in any tissue it comes in contact with [1]. Pure capsaicin is a hydrophobic, colorless, odorless, and crystalline-to-waxy compound and acts as a powerful stimulant of local heat receptors in mucous membranes and the skin. Capsaicin creates a sensation of warmth in the mouth and swallowing of sufficient doses causes severe pain. The most marked effects are evident in the peripheral part of the sensory nervous system and the efferent fiber [2]. Consequently, it is reported to mediate both immediate and chronic effects on the nerves. The specificity and low thresholds led to its frequent use as an experimental tool in neurophysiology [2,3]. Human environmental and dietary exposure to capsaicin is ubiquitous. Adults are mainly affected by capsaicin in a range of tolerable nociceptive perception via taste or touch unless they accept large concentrations that cannot maintain equilibrium in the body [4]. This effect can be interpreted as the perception of the body’s instinctive sensation to protect it from external harmful stimuli. However, the implications of pregnant women being exposed to consecutive capsaicin by continuous consumption of spicy food or using the pain-relieving patch should be considered. According to a previous report, no fetal malformations or variations were observed in animal experiments in the presence of capsaicin, but a few studies demonstrate the effects of capsaicin on neonates who have almost completed the organization of their body (in terms of physiology) [2,4]. The focus of previous studies has been examined of aspects or mechanisms of induction of neuropathic pain through topical administration of capsaicin in adult animals [5,6,7]. The transient receptor potential vanilloid 1 (TRPV1) channel is activated by capsaicin, and known as the transducer of chemical stimulation and a transmitter of nociceptive signals [8]. As the mechanism of function mediated by TRPV1 in the regulation of body temperature has been newly established, there is an opportunity to broaden the field of observation toward physiological changes [9]. In the process of conducting various neurophysiological studies on TRPV1 by administering capsaicin immediately after birth to neonates, various interesting changes in the physiological functions have been reported [10,11,12,13,14,15,16,17,18]. Accordingly, the unique physiological changes that may occur due to exposure to capsaicin to neonates are reviewed herein.

## 2. Neonatal Capsaicin Treatment and TRPV1

TRPV1 is a non-selective cation channel that can be activated by various exogenous and endogenous physical and chemical stimuli [19]. TRPV1 is found mainly in the nociceptive neurons of the peripheral nervous system and involved in the transmission and modulation of nociceptive sensation as well as other diverse painful stimuli [19,20]. It is known to be the target of capsaicin, the active component of chili peppers, and it can be referred to as the capsaicin receptor [21]. When TRPV1 is activated by capsaicin, sodium and calcium ions flow through TRPV1 into the cell to depolarize the nociceptive neurons [21]. The activation mechanism of TRPV1 using capsaicin has been reported in various studies [22,23]. Depending on the duration of capsaicin exposure, the calcium influx triggered by TRPV1 channels desensitizes the channels, thereby representing a feedback mechanism that protects the nociceptive neuron from toxic calcium overload [22]. This enables investigation of the mechanisms that underlie the acute capsaicin-induced desensitization of TRPV1 channels and allows us to explore capsaicin-induced analgesia [22]. However, this desensitization accounts for most of the reduction in responsiveness that occurs initially (within 20 s) after the first administration of capsaicin [23]. Thus, numerous studies on various sensory mechanisms mediated by TRPV1 have been conducted via the single or repetitive treatment of capsaicin in adult rodents [5,6,7]. Meanwhile, a few studies reported that single dose of subcutaneous injection of capsaicin into the newborn rat leads to involuntary life-long TRPV1 desensitization in the spinal dorsal root ganglia [10,11,19]. The aftermath of neonatal capsaicin treatment reveals a continued decrease in the expression of the TRPV1 channel, including the mRNA level even several weeks after birth [10]. Consequently, it is apparent that capsaicin treatment to the neonatal rodents easily induces TRPV1 desensitization without the hassle of repetitive capsaicin administration, and the method enables provable observation of a variety of physiological changes related to TRPV1 desensitization. Table 1 lists the studies on sensitization and/or desensitization of neurons by topical or neonatal capsaicin treatment [5,6,7,11,22].

## 3. Loss of Pain Perception

Capsaicin exerts its major pharmacological effects on the peripheral part of the sensory nervous system, particularly on the primary afferent neurons of the small unmyelinated (C-fiber) type [24]. Due to its potential as a specific toxin for peripheral C-fiber, capsaicin has been used extensively as a versatile tool in the study of pain mechanisms [10,12,25,26]. TRPV1 is expressed in a substantial proportion of C-fiber neurons, and its activation by capsaicin affects the function of C-fiber [27,28]. Parental administration of capsaicin to neonatal rats resulted in the permanent destruction of small B type sensory ganglion cells and of 80–95% of afferent C-fiber in the lumbar dorsal roots [24,29]. In the saphenous nerve, which has afferent and sympathetic efferent C-fiber, a 60–70% loss of C-fiber was observed [29]. This massive loss of afferent C-fiber is thought to result in the deficit of nocifensive reflexes, such as the reflex to a noxious stimulus. Various metrics have been used to investigate the pain response in neonatal capsaicin-treated rats, including the tail-flick and hot plate test that are used to determine thermal thresholds, the paw pressure test that is used to determine mechanical thresholds, and the formalin test that is used to determine chemical thresholds [30,31]. The tail-flick test is used in basic pain research to measure the effectiveness of analgesics by observing the reaction to heat. Generally during the test,, an intense light beam is focused on the animal’s tail, and the elapsed time or latency before the tail is moved is a measure of its pain threshold [30]. The paw pressure test is a technique for the measurement of the pain response in animals and is used to test the effectiveness of analgesics by observing the reaction to gradually increasing pressure on an inflamed paw [32]. The formalin test is an experimental assay of the licking and flinching response after the subcutaneous injection of formalin normally in the plantar hind paw. Formalin induces a nociceptive response lasting for 45–90 min [31]. It has been demonstrated that capsaicin treatment in neonatal rats caused analgesia to the representative types (thermal, mechanical, and chemical) of nociceptive stimuli examined depending on the destruction of the nociceptive fiber [10,12,30]. Further, antinociceptive effects following neonatal capsaicin treatment to noxious chemical and mechanical stimuli follow a similar trend [30]. As TRPV1 (expressed in a significant proportion) in C-fiber neurons is decreased by neonatal capsaicin treatment, the degustation caused by noxious taste also gets affected by neonatal capsaicin treatment [10]. This is since capsaicin binds with pain receptors in the mouth and throat, which are normally responsible for sensing noxious heat and sending pain signals [33]. To assess the ability to consume capsaicin water, intake volume was measured using a paired preference test [10]. Naïve or neonatal capsaicin treatment rats were given free access to capsaicin-dissolved and distilled water. The naïve rats preferred vehicle water rather than capsaicin containing water. The neonatal cap-treated rats did not show a preference between capsaicin water and vehicle water [10]. The neurotoxic action of capsaicin was observed along with reproducible and consistent depletion of primary afferent fiber that play a unique role in pain recognition. These analgesic effects indicate that variability in nociceptive thresholds is a fundamental and inherent principle behind the elimination of certain primary afferent fiber at an early developmental stage [33,34]. They may also indicate the complex nature of the changes that may occur in primary afferent connectivity or the central nervous system after neonatal capsaicin treatment [10,12,30]. However, research on this connectivity has not advanced very far. Table 2 lists the reported studies on pain related to topical or neonatal capsaicin treatment [10,12,25,26,30].

## 4. Changes in Body Temperature and Exposure to Infection

TRPV1 is expressed in a subset of peripheral and central neurons where it mediates various sensory functions including thermosensation and is directly activated by heat and compounds that modulate sensations of noxious hot temperature [9,35]. Therefore, TRPV1 is considered a vital part of the investigation of the molecular mechanisms for the detection of noxious heat above 42 °C [35]. Consistent with this thermoregulatory role, capsazepine (TRPV1 antagonists) has been reported to reduce heat sensitivity and elevate body core temperature, whereas an acute drop in body core temperature (hypothermia) can be induced by administration of capsaicin [36]. It is a defense mechanism against noxious heat reactions caused by harmful stimuli such as capsaicin and aims to maintain body temperature homeostasis [37]. Suppression of the activity of TRPV1 through an antagonist decreases the sensitivity to heat and induces malfunction in the body’s ability to sense high temperature [36]. Rats who received capsaicin systemically within 48 h of birth showed continuously decreased TRPV1 expression after the only one administration [10,11,19]. The neonatal capsaicin-treated rats showed an increased body core temperature (hyperthermia) similar to the TRPV1 antagonists-treated rats [10,36]. The difference between TRPV1 antagonist and neonatal capsaicin treatment is that the hyperthermic effect of capsaicin treatment in neonates is long-lasting [10]. Previous studies on body temperature changes following topical or neonatal capsaicin treatment are listed in Table 3 [13,38,39,40,41]. Numerous studies have been carried out on the analysis of the effect of hyperthermia on immune cell subsets targeting the tumor, and upregulation of interleukin induced T lymphocyte proliferation by TRPV-1 activation was found to be increased by hyperthermia [42]. It is known that the systemic activation of the immune system might contribute towards targeting the metastatic tumor cells during hyperthermia [43]. Also, the effects of TRPV1 may be seen in the context of respiratory diseases [44,45]. Activation of TRPV1 expressed in sensory neurons leads to inflammatory diseases, such as bronchoconstriction, tracheal mucosal edema, and inflammatory cell chemotaxis [45]. A related study on neonatal capsaicin treatment indicated that induction of constant and permanent hyperthermia in the body might induce an abnormal physiological function related to the body defence system [13]. Brown adipose tissue present in human and animal newborns is responsible for maintaining body temperature and is essential for thermogenesis. Brown adipose tissue contains numerous cell types in addition to monocytes and macrophages thereby relating its critical role in mediating immune response [46,47]. Brown adipose tissue is rapidly lost postnatally, and this process gets concluded within the first few years of life. However, the volume of brown adipose tissue increased more as neonatal capsaicin-treated rats grew into adults [13]. Although the mechanism has not been clarified yet, it is anticipated that the abnormal phenotype is mediated by the downregulation of TRPV1 followed by the overload of thermoregulation activity in brown adipose tissues [13]. Leptin contributes to cutaneous antimicrobial defense system by upregulating the expression of defensins [48]. Abnormality in brown adipose tissue function affects leptin secretion, and its expression has been reported to be significantly reduced in neonatal capsaicin-treated rats [13]. It is hypothesized that leptin–associated decreased expression levels of beta-defensin in neonatal capsaicin-treated rats might have initiated immune dysfunction, thereby leading to a decline in the host defense from bacterial infections. Although there is only one report on an increase in the infection level in neonatal capsaicin-treated rats via abnormal function of brown adipose tissue, there are two more studies examining the relationship between neonatal capsaicin treatment and changes in the level of infection. An extensive level of infection of glial cells proximal by amplified degenerative effect on central branches of the substance P in capsaicin treated neonatal mice has been reported [15]. Substance P provides protection against viral, bacterial, and parasitic infections. A significant increase in mycoplasma pulmonis infection caused due to a decrease in the immunoreactivity of substance P by neonatal capsaicin treatment has been reported [14]. Vulnerable exposure to various sources of infection due to lack of substance P is the degenerative effect of the sensory nerve following neonatal capsaicin treatment, thereby causing a reduction in the secretion of substance P (Table 4).

## 5. Chronic Itching

Known triggers for pruritus include exposure to allergens pollen or foods), stress, dry skin, and infection [49]. Neonatal capsaicin-treated rats are known to be vulnerable to infection [13]. A previous study consistently reported that all the treated animals had long-lasting, occasionally relapsing, and severe cutaneous lesions over the entire body and that these lesions were closely related to vigorous scratching behavior [16]. The phenomenon was similar to behavioral patterns and symptoms were observed in association with atopic dermatitis [16]. Rats administered with capsaicin at the neonatal stage began to show scratching behaviors from 2 or 3 weeks of age and lesions with flare, bleeding, and ulceration appeared on the skin around the face and ears at 4 weeks of age [16]. The following is a summary of the symptoms observed in terms of histopathological and molecular biological changes: cutaneous nerve fiber were significantly reduced in capsaicin treated neonatal rats compared to naïve animals, indicating an irreversible loss of cutaneous nerve fiber due to neonatal capsaicin treatment [16]. Filament aggregating protein (filaggrin) reactivity was completely abolished in the skin, despite the clear presence of epidermal hyperplasia [16]. All lesional skin samples were found to be colonized with a large number of *Staphylococcus aureus* [13,16]. Transepidermal water loss was remarkably higher in capsaicin treated neonatal rats than in the control [16]. Abnormal histopathological changes like epidermal hyperplasia, hyperkeratosis, and a large amount of mast cell infiltration were observed around the lesional skin [16]. Since TRPV1 is expressed in a substantial proportion of C-fiber neurons, it was hypothesized that neonatal capsaicin treatment could lead to permanent loss of cutaneous nerve fiber [10,11,19,50]. The majority of massive loss of nerve fiber is supposed to be peptidergic because most of the capsaicin-sensitive primary afferents contain neuropeptides, substance P, and calcitonin gene-related peptide [14,15,51]. It has been reported that calcitonin gene-related peptide-positive nerve fiber densely innervate the epidermis and upper dermis where calcitonin gene-related peptide stimulation may contribute to epidermal reconstruction by inducing proliferation of keratinocytes [52,53]. Filaggrin binds with keratin fiber in epithelial cells and is affected by the loss of nerve fiber or substance P. Considering that filaggrin is the main constituent of the skin’s barrier, decreased expression of filaggrin in the skin can cause increased susceptibility to bacterial infections [54]. Besides, an immune defense system that is under physiological stress due to hyperthermia might further exacerbate itching. Another underlying cause of pruritus induced by neonatal capsaicin treatment can be uncovered by focusing on the lack of pain perception [10,12,13,30,55]. Neonatal capsaicin treatment resulted in the permanent destruction of afferent C-fibers in the skin, thereby resulting in chronic itching. The reason is that they would not be sensitive to compensation stimuli, such as pain. Therefore, neonatal capsaicin-treated rats continue to suffer from chronic itch even though a normal rat would have impeded itching sensation due to the pain perception with scratching behavior [18]. Clinically, this is referred to as neuropathic itching. Relief of itch may come from an increase in molecules that can be suppressed and/or parameterized directly through the neurons in-charge, nevertheless these symptoms can be eliminated by compensation from other sensory neurons, like pain sensation [18,55]. Chronic itching is exacerbated by the lack of pain sensation signifying reduced compensation for scratching, so the desire to resolve itch purely through scratching is maximized [18,55,56]. It is almost impossible to constantly resist the urge to scratch caused by serious chronic itching, and scratching can even occur during sleep or be inadvertent [57]. Given that the approach to this symptom is only to screen for medical records of pre-disease conditions that exist before the onset of symptoms, a method employing capsaicin treatment in neonatal rats may be a useful tool for investigating the mechanisms of the neuropathic itch. The studies on itching caused by neonatal capsaicin treatment are listed in Table 5 [16,17,18].

## 6. TRPV1 and Circadian Body Temperature Rhythm

One particular study aimed to observe the effects of treatment with capsaicin on circadian rhythms and fortunately the investigators were able to draw constructive conclusions from the results reported by Jancso-Gabor in 1970 [38]. Local treatment with a large amount of capsaicin was used as a tool for directly destroying the hypothalamus, and all theoretical explanations were pursued from the perspective of neurobiology [38]. From this groundbreaking method, interesting results were reported by Satinoff in 1982 [58]. The report indicated that an increased amplitude in the circadian variation of body temperature in the locally treated rat with a large amount of capsaicin (medial preoptic area lesioned rat) was due to a rise in the upper and lower limit of body temperature [58]. Based on the theory of TRPV1 being a channel directly affected by capsaicin and that it has the function of thermoregulation related to noxious heat sensations, studies on tools to suppress the gene expression of TRPV1 were conducted in addition to studies on investigating the method of specific nerve damage following local treatment with capsaicin [35,59]. Further, investigations on various molecular biological factors involved in circadian rhythm were conducted along with studies on the intersection of the mechanism between TRPV1 and clock genes [10,60,61,62]. However, it is still unclear whether the channel regulates clock gene expression, which would define a novel pathway for the circadian rhythm modulation in peripheral tissues, in contrast, the role of TRPV1 in energy metabolism and thermogenesis has been well established, as described in the previous paragraphs. A recent paper reported on a malfunction in circadian rhythm for core body temperature with a focus on the phenomenon through which the expression of TRPV1 was downregulated [10,61]. In normal rodents, body temperature increases by about one degree at night compared with daytime because of their nocturnal behavior [10,61]. Only one case has been reported that the rhythm was reversed in the neonatal capsaicin-treated rats while it was consistent in naive rats [10]. Many putative clock genes including Period circadian regulator (Per) 1, Per2, Brain, and Muscle ARNT-Like protein (Bmal) 1 and Heat shock factor (Hsf) 1 are expressed in the hypothalamus and liver [63]. To ensure a systematic system rhythm for the whole organism, all tissue clocks must be aware of the same temporal environment and synchronize with each other [64]. Under normal physiological conditions, the expression of genes encoding positive regulators, such as Hsf-1 and Bmal1is at a peak in the daytime [65,66]. The expression of genes encoding negative regulators, such as Per1 and Per2, peak at the night contrary to Bmal1 expression [66]. In neonatal capsaicin-treated rats, expression of Per1 and Bmal1 was not altered, but Hsf1 and Per2 genes were abnormally expressed [10]. Hsf1 is not only in-charge of daily body temperature oscillations but also serves as a key transcription factor in heat response [65,67]. Therefore, Hsf1 could be regarded as a factor corresponding to a decrease in the expression of TRPV1, and this is consistent with an increase in hypothalamic Hsf1 expression in neonatal capsaicin-treated rats with hyperthermia [10,65,66,67]. In other words, the circadian rhythms are affected by hyperthermia, thereby changes in the expression of clock genes in the hypothalamus and liver disrupt daily circadian rhythm especially in terms of core body temperature [10,61]. Although many studies on the relationship between TRPV1 and clock genes have been conducted, only two studies are listed in Table 6 that investigated the circadian body temperature rhythm by topical application or neonatal capsaicin treatment [10,61]. Consequently, at the point where TRPV1 can be sensitized or desensitized through a variety of capsaicin-based methods, the potential for setting to right the complex networks for clock genes might be able to emerge with further study utilizing the neonatal capsaicin treatment.

## 7. Conclusions

A variety of physiological changes associated with neonatal capsaicin treatment are induced by desensitization of its direct target channel, TRPV1, suggesting that capsaicin has a broad effect on sensory and on all the homeostatic actions involved in TRPV1. It can be described as a unique phenomenon in which the reduction of pain perception, induction of hypo- or hyperthermia, increase in infection, infectious or neuropathic itching, and irregular circadian core body temperature rhythm are induced. Depending on these findings, it may warn several symptoms that arise due to capsaicin exposure in the neonatal period, further it should also be recognized that the listed physiological abnormalities belong to a rare disease that are unlikely to be experienced in normal. Further, based on the effect of capsaicin on TRPV1, which involves a complex interaction between neurophysiological and molecular biological mechanisms, it is clear that further exploration of the systematic action is needed. Furthermore, looking more closely at this unique phenomenon caused by systemic capsaicin administration in neonatal rats, it would be expected that clinical benefits applicable to the systematic exploration of various intractable diseases can be obtained.

## Figures and Tables

**Table 1:** Summary of studies on capsaicin-induced sensitization or desensitization of neurons.

Study By	Study Design	Subject	Measurement	Results
Simone et al. [6].	Intradermal injection of 100 µg of capsaicin	Cat	Innocuous mechanical stimulation	Sensitization of cat dorsal horn neurons
Donnerer et al. [5].	Daily subcutaneous injection of 50 mg/kg of capsaicin	Rat	RT-PCR	Desensitization of TRPV1 in dorsal root ganglia
Vyklicky et al. [22].	Treatment with 1 µM of capsaicin	HEK293T cells transfected with rat TRPV1	Whole-cell current responses	Selectively excite and subsequently desensitize nociceptive neurons
Srbely et al. [7].	45 degrees heat for 10 min then received topical capsaicin cream (0.075%)	Human	Central sensitization at the C(5) segment and mechanical cutaneous sensitivity	An increase in central sensitization related to allodynia
Newson et al. [11].	0.1 M (5 mg/mL) neonatal capsaicin treatment	Rat	Superior staining for neuronal counting	Life-long loss of sensory neurons expressing TRPV1 channels.

**Table 2:** Summary of studies on capsaicin-induced desensitization of pain perception.

Study By	Study Design	Subject	Measurement	Results
Meller et al. [12].	Neonatal capsaicin treatment (50 mg/kg)	Rat	Thermal hyperalgesia in a sciatic nerve ligation model	Loss of thermal hyperalgesia
Dougherty et al. [26].	Intradermal capsaicin administraion (0.1 mL, 1% solution)	Monkey	Cutaneous mechanical and thermal stimuli by an electrode	Loss of thermal and mechanical stimuli
Baamonde et al. [25].	Intra-plantar administration of 10 μg of capsaicin	Mouse	Licking behavior following complete Freund’s adjuvant injection	Induction of long-lasting analgesia for at least 2 weeks
Jeong et al. [10].	Neonatal capsaicin treatment (50 mg/kg)	Rat	Paw-withdrawal latency to radiant infrared heat stimulation, and intake volume of capsaicin water	Desensitization to noxious heat stimuli and impaired sensing of capsaicin
Nagy et al. [30].	Neonatal capsaicin treatment (5 to 100 mg/kg)	Rat	Noxious thermal, mechanical, and chemical stimuli	Increased nociceptive threshold by higher doses of capsaicin, but the uncertain correlation with the extent of loss of primary afferent fiber

**Table 3:** Summary of studies on capsaicin-induced body temperature changes.

Study By	Study Design	Subject	Measurement	Results
Jancso-Gabor et al. [38].	Subcutaneous injection of capsaicin (0.25 mg)	Rat	Rectal temperature	A decrease in the rectal temperature for 120 min
Cabanac et al. [39].	Subcutaneous injection of capsaicin (6-66 mg, cumulative)	Rat	Rectal temperature	Induction of hyperthermia by a decreased salivary secretion
Jeong et al. [13].	Neonatal capsaicin treatment (50 mg/kg)	Rat	Core body temperature	Induction of chronic hyperthermia
Mori et al. [40].	Intragastric administration of capsaicin (10 and 15 mg/kg)	Mouse	Colonic temperature	Decreased colonic temperature by 15 mg/kg capsaicin, and increased colonic temperature by but 10 mg/kg capsaicin
Inagaki et al. [41].	Oral gavage of capsaicin (10-20 mg/kg)	Mouse	Core body and tail surface temperature	A decrease in core body temperature and an increase in tail surface temperature

**Table 4:** Summary of studies on the relationship between neonatal capsaicin treatment and infection.

Study By	Study Design	Subject	Methods	Results
Ljungdahl et al. [15].	Neonatal capsaicin treatment (50 mg/kg)	Mouse	Herpes simplex virus infection	The extensive infection of glial cells proximal by an amplified degenerative effect on central branches of the substanceP
Bowden et al. [14].	Neonatal capsaicin treatment (50 mg/kg)	Rat	Mycoplasma pulmonis infection	Severe infection in the airway mucosa by reduction of substance P immunoreactive nerve fiber
Jeong et al. [13].	Neonatal capsaicin treatment (50 mg/kg)	Rat	Staphylococcus aureus infection	An increase in the Staphylococcus aureus infection by disruption of the immune defense system

**Table 5:** Summary of studies on itching following neonatal capsaicin treatment.

Study By	Study Design	Subject	Measurement	Results
Back et al. [16].	Neonatal capsaicin treatment (50 mg/kg)	Rat	Scratching behavior and dermatitis score	Severe scratching behavior and cutaneous lesions by loss of immunoreactions in sensory nerve
Thomas et al. [18].	Neonatal capsaicin treatment (50 mg/kg)	Rat	Damaged skin and behaviors of scratching	Induction of scratching behavior resulting in the total area of skin damage by loss of c-fiber
Jeong et al. [17].	Neonatal capsaicin treatment (50 mg/kg) + juvenile obesity	Rat	Dermatitis score	Robust development of dermatitis by juvenile obesity

**Table 6:** Summary of studies on changes in circadian body temperature rhythm by capsaicin treatment.

Study By	Study Design	Subject	Results
Szelényi et al. [61].	Subcutaneous injection of capsaicin (15 and 30 mg/kg) on two consecutive days	Mouse	Higher daily body temperature amplitude in the capsaicin pretreated mice by vanilloid receptor-mediated altering action on temperature regulation
Jeong et al. [10].	Neonatal capsaicin treatment (50 mg/kg)	Rat	The reverse amplitude of the circadian body temperature rhythm in neonatal capsaicin treatment rats caused by altering the expression of clock genes in the hypothalamus and liver

## Abbreviations

Bmal1	Brain and Muscle ARNT-Like protein 1
Hsf1	Heat shock factor 1
Per1	Period circadian regulator 1
Per2	Period circadian regulator 2
TRPV1	Transient receptor potential vanilloid 1
